# A functional approach to movement analysis and error identification in sports and physical education

**DOI:** 10.3389/fpsyg.2015.01339

**Published:** 2015-09-10

**Authors:** Ernst-Joachim Hossner, Frank Schiebl, Ulrich Göhner

**Affiliations:** ^1^Institut für Sportwissenschaft, Universität BernBern, Switzerland; ^2^Institut für Sportwissenschaft, Eberhard-Karls-Universität TübingenTübingen, Germany

**Keywords:** movement science, task analysis, constraints, augmented feedback, dynamical systems, internal models, modularity, basic action concepts

## Abstract

In a hypothesis-and-theory paper, a functional approach to movement analysis in sports is introduced. In this approach, contrary to classical concepts, it is not anymore the “ideal” movement of elite athletes that is taken as a template for the movements produced by learners. Instead, movements are understood as the means to solve given tasks that in turn, are defined by to-be-achieved task goals. A functional analysis comprises the steps of (1) recognizing constraints that define the functional structure, (2) identifying sub-actions that subserve the achievement of structure-dependent goals, (3) explicating modalities as specifics of the movement execution, and (4) assigning functions to actions, sub-actions and modalities. Regarding motor-control theory, a functional approach can be linked to a dynamical-system framework of behavioral shaping, to cognitive models of modular effect-related motor control as well as to explicit concepts of goal setting and goal achievement. Finally, it is shown that a functional approach is of particular help for sports practice in the context of structuring part practice, recognizing functionally equivalent task solutions, finding innovative technique alternatives, distinguishing errors from style, and identifying root causes of movement errors.

## Setting the stage: Performance errors and a functional framework

When it comes to the identification of performance errors in sports and physical education, on the teacher's or coach's side, a well-grounded idea is demanded on what kind of performance counts as an error and what kind of performance does not. Thus, the question arises on how this idea can be underpinned. In this regard, it seems useful to distinguish, in the first place, errors in decision-making from errors that are related to the movement as a means of putting the decision into practice. Failure can result from both because a good realization of a bad decision is to the same degree worthless as a bad realization of a good decision. In the following, only performance errors of the second kind will be considered further, meaning that the pursued perspective will be less rooted in the psychology of decision-making but more in a movement-scientific framework of motor control and learning. Hence, for the issue at hand, the scope can be narrowed down in such a way that independent from the quality of decision-making, a well-grounded idea is demanded from the teacher's or coach's side on what kind of motor performance counts as an error and what kind of motor performance does not.

As it should become obvious over the following text, a functional approach to movement analysis provides a valuable tool for practitioners who are faced with exactly this problem of error identification. The respective discussions are based on decades of work in German sport science conducted by the third author. However, in the paper at hand, the functional approach is presented to an international audience for the first time. Furthermore, the approach will be enriched by links made between the approach-specific starting point of the analysis of movement tasks and mechanisms that underlie the movement generation on the level of motor control. Hence, from a motor-control perspective, this paper should also be understood as a contribution to relating psychological, internal control structures to the achievement of goals in the physical, external world.

When trying to identify errors in motor performance, unfortunately, this task turns out to be trivial in rare cases only. Error identification is easy if, for instance, well-defined rules are broken, for example, in track-and-field's long jump, the foul line at the end of the board is crossed by the jumper or, in tennis, the serve does not hit the service box. However, even in those cases of easily detectable errors, the coach is expected to come up with feedback that does not directly refer to these errors because advices in the form of “hit the board” or “hit the box” do obviously not help. Instead, the coach's task would be to identify the root cause of the error. This cause can rarely be found outside the athlete's sphere of influence as it true, for example, in an unpredictable squall in ski flying or an “impossible” opponent's dig in beach volleyball. In the vast majority of cases, however, the error is caused by the athlete him- or herself. Consequently, the coach is supposed to search for reasons why a particular action missed, for example, by not hitting the board or the service box. Such athlete-related reasons might be found in a suboptimal execution, for instance, of the last steps before a jump or in an insufficient ball-toss height for the service.

Hence, even in these trivial examples, performance errors have to be identified with respect to the athlete's motor performance. As a matter of course, the same is true if rules are not broken but the resulting performance does not lead to a desired outcome, which means, that the jump was valid but not long enough to achieve a better rank or that the serve hit the service box but could easily be returned by the opponent. If, in those cases, the unsatisfying outcome cannot be attributed to current limitations in physical or coordinative respect, the resulting performance has to be counted as underachievement, and thus, as an error made by the athlete him- or herself. Consequently, the coach is again in charge of finding the error's root cause.

Generally, an error can be defined as a deviation from a specific target. Thus, the teacher or coach needs to access two kinds of measures: the actual value, specified by the movements as they are actually performed by the athlete, and the desired value, defined as the respective movements as they optimally look like. At this point, it should be underlined that *both* values are often not easily gathered. In this respect, the analysis of the actual performance can be a hard task by itself, particularly in sports where movements are executed with high speed as it is true in fencing or ski jumping. In those cases, when solely relying on one's own perception, it might be helpful for the coach to focus on crucial aspects of the athlete's movement repeatedly, maybe supported by the choice of an optimal angle of observation or by the pre-definition of a perception-enhancing gaze strategy. In addition, the coach could apply video technology, or if available, count on even more elaborate measures derived from biomechanical analyses. However, as this paper focuses more on the conceptual understanding of movement errors and less on their diagnosis on a technical level, we want to assume for the moment that the access to the actual movement value is not a problem. Then, the overall issue of error identification is reduced to the availability of a desired value.

Regarding the identification of desired values, two kinds of approaches can be used in sports practice. The first approach is based on the performance of top-level athletes, and thereby, on the idea that peak performance necessarily comes along with the highest level of movement-related expertise. Consequently, in textbooks on sport-specific didactics, techniques are often illustrated by a series of pictures taken from an international champion in order to give the reader an idea of how the respective movement should ideally be executed.

However, this approach is infected with a number of issues. First, even in top-level sports, elite athletes differ considerably regarding details of their movements. On an expert level, those individual features are typically referred to as “personal style.” At this point, for the topic at hand, the issue arises on how style-related and non-style-related features can be distinguished from each other if the description of the performed movements is, as presumed above, the sole basis for the definition of the desired value. Secondly, it makes sense to assume that style-related features are mainly due to differences in the athletes regarding their physical or physiological conditions. Consequently, in basketball, for instance, for athletes of different body heights, masses or leg muscle strengths, the desired values for the jump to a layup should also be expected to differ. Apparently, this issue is further increased if athletes of different levels of expertise are compared to each other, for instance, an adult, tall, highly trained top-level athlete depicted in a textbook's picture series with a young, small and weak schoolchild who needs the teacher's advice for learning the basketball layup. In those cases—that are absolutely typical for physical education as well as for sports practice below a top-level threshold—it seems questionable whether mere illustrations and descriptions of elite athletes' performances should be considered as helpful at all.

The second approach, which in our eyes is the superior approach to the identification of movement errors, can be labeled as “functional” as desired values are not based on experts' movements but on the functions that are fulfilled by the movement or by certain parts of the movement. Thus, this approach aims on a deeper understanding of the task the athlete is confronted with by trying to find answers to the question of what a certain movement characteristic is good for. This objective, in turn, implies a reorientation away from performed movements toward a focus on individual goals the performer strives for by means of appropriate movements. Hence, from a functional perspective, movements can be regarded as more or less functional with respect to the achievement of desired goals. For this reason, a defining feature of a functional approach can be found in the imperative to connect movement characteristics to goal-related purposes. Consequently, the description of a movement characteristic is typically combined with a subordinate clause beginning with “in order to” that provides the recipient with an explanation for what reason the respective movement aspect is of particular use.

As illustrated in Table 1 by a couple of examples taken from track-and-field athletics, a biomechanical perspective turns out to be of particular heuristic value when it comes to the functional substantiation of movement characteristics. This seems to be the case as movements are generally marked by positional changes of physical bodies in space over time. In this regard, functional substantiations may refer to the optimization of the acceleration path, the transfer of energy between body parts, the exploitation of the action-reaction principle as well as a range of others.

**Table 1:** **Biomechanically substantiated characteristics of sports movements**.

**Movement**	**Perspective**	**Movement characteristic and functional substantiation**
Shot put	Biomechanical	The athlete begins a trial bend forward and facing the rear of the circle *in order to* maximize the length of the path that, under the spatial limitations defined by the circle, can be exploited for the acceleration of the shot
Javelin throw	Biomechanical	The athlete accelerates legs, trunk, shoulder, and arm in succession *in order to* increase the velocity of the javelin by successively transferring energy from the lower to the upper body parts
Long jump	Biomechanical	The athlete prepares landing by bringing the feet (and, due to the action-reaction principle, at the same time arms and upper body) forward *in order to* maximize the distance defined by the nearest impression made in the pit
High jump	Biomechanical	The athlete crosses the bar backwards in an arched position with legs and shoulders hanging down *in order to* achieve a measured height which is, related the maximum height of the body's center of mass, as large as possible
100-m sprint	Biomechanical	The athlete starts from the blocks at a low angle *in order to* guarantee that the first accelerating steps can be performed with a minimum of horizontally acting braking forces
50-km race walk	Biomechanical	The athlete supports the steps by moving the pelvis back- and forward *in order to* maximize the propulsion in forward direction whilst obeying the rules on a permanent contact to the ground and a straight leg in the cycle's first phase

However, note that a functional approach to the specification of desired values is not restricted to substantiations drawn from a biomechanical perspective. Instead, as illustrated in Table 2, by a range of examples from different sports, reasonable functional explanations can also be derived from anatomical, physiological, coordinative, perceptual, mental, or tactical perspectives on the performed movement, and the same could be true for further domains that are not explicitly addressed in Table 2. The reason for those kinds of functional substantiations can be found in the fact that although movements, in the end, are defined by biomechanical measures—these movements are realized by the use of body systems by a human performer who has to control actions on the basis of perceptual-motor capabilities and psychological competences. Thus, it may be that biomechanical constraints are “overwritten” by non-biomechanical effects that turn out to be more decisive for the achievement of an optimal motor performance.

**Table 2:** **Non-biomechanically substantiated characteristics of sports movements**.

**Movement**	**Perspective**	**Movement characteristic and functional substantiation**
Barbell half-squats in strength training	Anatomical	When performing the repetitions with high loads, the athlete keeps his or her back straight *in order to* prevent the backbone from injuries resulting from repeated overload
Downhill ski racing	Physiological	When facing a bumpy slope, the athlete modulates muscle stiffness *in order to* absorb the perturbations by making use of the spring characteristics of the muscles
Blocking in volleyball	Coordinative	The athlete (typically) refrains from a biomechanically optimal full arm swing *in order to* facilitate the coordination of the arm movement into the opponents' space as close to the top of the net as possible
3-m springboard diving	Perceptual	After multiple rotations, the athlete focuses on certain landmarks, for instance, at the wall of the bath, *in order to* facilitate visual orientation in space that is needed for an optimal entry
Basketball free throw	Mental	The athlete (typically) performs a pre-shot routine, for instance, by bouncing the ball twice, *in order to* shield his or her attentional focus against distractions (e.g., against noise made by spectators)
Football penalty kick	Tactical	The athlete (typically) refrains from a biomechanically optimal approach *in order to* hide his or her intention regarding the direction of the strike from the goalkeeper

The examples listed in Table 2 are also helpful to explicate two further features of the functional approach to movement analysis. First, the strength-training example shows that crucial functions are not necessarily restricted to effects that immediately result from movement execution but may also refer to consequences in the far future. Second, as illustrated by the springboard-diving example, intended movement effects do not necessarily refer to effects that are achieved by means of the movement but may also refer to the movement itself. Such a movement-relatedness of the action goal can be particularly observed in sports that are marked by evaluations given by judges. Besides springboard and high diving, typical examples for such a coincidence can be found in gymnastics, figure skating, synchronized swimming, dressage, or competitive dancing. Accordingly, in gymnastics, for instance, stretched feet are not functional with respect to the successful performance of a front scale, the main reason for stretching the feet rather regards the fact that the fulfillment of this criterion is a prerequisite for the achievement of high judgments. Consequently, those judgment-related aspects of the movement should also be accepted as “in order to” substantiations to get the whole world of sports covered by a functional approach to movement analysis.

## Functional movement analysis: Constraints, sub-actions, modalities, and functional assignments

The approach of a functional movement analysis can be traced back to Ulrich Göhner, who originally published the concept in the 1970s (Göhner, 1974, 1979) and refined it further over the subsequent decades (Göhner, 1992). In its current form, the approach is labeled best as “action-oriented functional movement analysis” because it is not the movement itself but the acting athlete who forms the starting point for the analysis (Göhner, 2013). Consequently, movements are understood as solutions of tasks that are posed to an athlete.

In a nutshell, a functional movement analysis can be broken down into the four steps of (a) considering constraints that affect the to-be-performed movement task, (b) identifying sub-actions that underlie the observable movement, (c) specifying, if needed, these sub-actions by determining modalities for an optimal execution of the respective movement part, and (d) ascribing functional assignments to sub-actions and modalities in order to accomplish a complete functional understanding of the movement at hand. In the following, these steps will be discussed in more detail.

### Constraints

**Figure 1:**
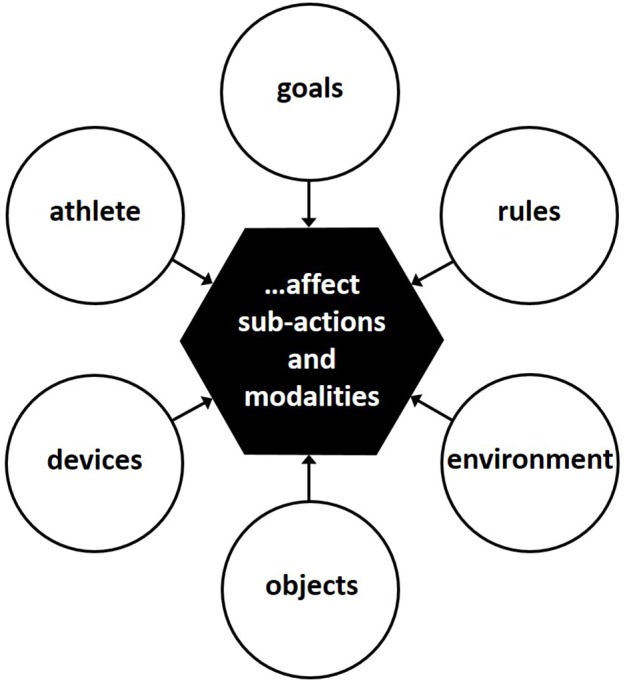
**Constraints affecting the identification of a desired value of a sports technique**.

If movements are first and foremost understood as the means for the solution of motor-related tasks, then the first logical step for a functional analysis refers to gathering knowledge on all the characteristics that define the task to a remarkable extent. As summarized in Figure 1, these characteristics can be assigned to constraints regarding movement goals and rules as well as regarding specifics of the environment, of the to-be-moved object, of supporting devices or of the athlete him- or herself.

- *Goals* need to be considered because movement tasks can solely be defined as tasks for someone who intends to achieve a certain movement-related goal. So if the goal of a skier was to maximize control over the skis, a completely different movement should be expected than for a skier who just loves experiencing nature and thus strives for a minimization of effort. In the first case, a pronounced edging of the skiers is functional, in the second case, due to the increased effort, such a pronounced movement execution is non-functional. Common goals in sports refer to time minimization (e.g., ski slalom), distance maximization (e.g., shot put), hit optimization (e.g., fencing), error minimization (e.g., gymnastics), and difficulty maximization (e.g., figure skating). Apart from these objectives focussing on performance comparisons between athletes or teams, further common goals in sports are achievement-related like performing a difficult skill (e.g., acrobatics), maintaining tough states (e.g., surfing), or mastering a motor-related challenge (e.g., hillwalking). Finally, goals may also be rather unspecific to the situation at hand as it is the case in objectives regarding fitness, health, well-being, or keeping in touch.
- In the majority of cases, *rules* are officially defined by the respective international association. However, one has to keep in mind that in informal, non-official settings at least, rules may be negotiated as it is, for instance, quite common for the disregard of official pitch measures or the offside rule in children's football play. Besides, rules may be changed not only in informal settings but also officially as it became true for the somersault technique in track-and-field's long jump that had been compliant until it was explicitly banned by the rule commission. Thus, performing a somersault over the flight phase of a long jump might, from a biomechanical viewpoint, be the best solution for the task to maximize the perpendicular distance from the take-off board to the nearest break in the landing area, as soon as this technique had been banned, the solution was not functional anymore.
- Specifics of the *environment* particularly come into play in outdoor sports. For instance, in many water sports, the time needed to cover a predefined distance has to be minimized. Hence, the water as the sport-specific medium for performing a task is a prerequisite for swimming, canoeing, rowing etc.; however, due to the increased resistance of water in comparison with air, the medium also serves as a specific constraint as speed fluctuations are punished much harder. Comparable specifics can be found in other outdoor sports that are conducted on snow (e.g., downhill racing), on ice (e.g., bobsleighing), in the air (e.g., skydiving) or in other particular environments (e.g., parcours). Furthermore, in a number of sports, demand-increasing and unpredictably changing conditions (e.g., white-water rafting) are the major incentive for the athlete whilst in other sports, environmental effects are minimized, as it is true in standardized indoor sports like gymnastics, swimming, handball, or judo.
- Specifics of the *object*, quite trivially, are crucial whenever objects are involved in the motor task. In this respect, for instance, in discus throwing, due to the specific flight quality of the disc, it is important to release the disc with a certain rotation and at a certain angle with respect to the horizontal plane. In turn, the physical features of the object define whether a specific movement can claim to be functional or not. Quite obviously, the same is true for balls, darts, Frisbee discs, and air-rifle munition, in a nutshell, for all sports in which an object has to be moved as far or as precisely or as close to a pre-defined target or flight curve as possible. Less obviously, it can also be an opponent who has to be moved as it is the case in wrestling, in rope pulling, or, when striving for a knockout, in boxing. Even less obviously, very often, the object coincidences with the movement-inducing subject, that means, with the athlete him- or herself.
- In many sports, the athlete is supported by specific *devices* that are needed in order to successfully perform the task to be solved. Consequently, in skiing, car racing or tennis, for instance, the overall performance is not only determined by the athlete him- or herself but also by the supporting material as well as by the degree the athlete is able to adapt his or her movements to certain specifics of the skis, the car or the racket. In those cases, the determination of functionally optimal task solutions also needs to consider specifics of the supporting devices.
- Finally, movements are functionally constrained by attributes of the *athlete* him- or herself. In this regard, for instance, the athlete's body height, his or her coordinative competence, peculiarities of the leg muscles' strength as well as other athlete-related factors might give rise to different optimal movement solutions of the task at hand. Furthermore, the functional analysis should also take into account whether the movement has to be executed with a teammate (e.g., synchronized trampolining or ballroom dancing), against an opponent (e.g., table tennis or wrestling) or without any interaction with other humans (e.g., mountain biking or walking).

### Sub-actions

After having gathered a deeper understanding of the task at hand by considering the role of constraints, the second step of a functional analysis would regard task solutions that are typically executed in sports practice. At this point, due to the fact that functions are defined by certain goals that have to be achieved by means of movements, the athlete not only comes into play as a constraint as explained above; the athlete is rather regarded as an actor who strives for achieving a desired outcome. For this reason, the second step of the functional analysis is driven by the question of which kind of sub-actions is performed by athletes in order to achieve the overall goal at hand.

First, an overall action goal should be identified on a medium grain-size level. This means that it neither seems to be appropriate to search for goals on the large or high level of “winning a match” or “sustaining health” nor does it make sense to seek goals on the small or low level of “lifting the right foot for the next step” or “bending the arms to prepare for an underhand-pass.” The high-level goal is not specific enough to be related to certain movements, while for the low level, the goal is not related to an action that can stand alone as a meaningful unit. Instead, the basic goals should refer, on the one hand, to concrete parts of the observable motor behavior that, on the other hand, still make sense when being considered in isolation. In the language of sports practice, these criteria are typically met by parts that are referred to as “techniques.” Examples for actions in such an understanding would be the glide technique in track-and-field's shot put, the flip-turn technique in swimming, the long-hand kip technique in artistic gymnastics, or the deep-spiral technique in paragliding. In all of these cases, the techniques are characterized by well-defined initial states, well-defined final states, and well-defined movement trajectories in-between. Furthermore, the techniques come along with meaningful overall goals, in the examples above, of maximizing the put distance, minimizing the time needed for the inversion of the swimming direction, executing the kip movement in an optimal form, and losing flying height as quickly as possible.

In continuously performed sport tasks, it may be a little bit harder to identify basic units for a functional analysis; however, in those sports, it makes sense to reduce the analysis to a single movement cycle, that means, for instance, in running, from the moment the right foot leaves the ground until the moment the right foot leaves the ground for the next time, or, in skiing, from the moment a turn to the right is initiated until the moment a turn to the right is initiated for the next time.

Beyond, the identification of overall action goals, from a functional perspective, it also makes sense to break the functional analysis down to sub-actions which are subordinate with respect to the overall action, but nevertheless, make an important contribution to the achievement of the desired outcome. Again, those sub-actions need to be connected to certain sub-goals in order to fit a functional framework. However, the sub-goals need not be directly related to the overall goal; instead, the sub-goal may also be determined with respect to the optimal execution of another sub-action that in turn, directly regards the overall aim of the action. In sports, those auxiliary functions can typically be found in the preparatory phase of a movement, for example, in the approach of a long jump, in the backswing of a golf stroke, or in the elevation of the body's center of mass at the beginning of a front-hip circle at the horizontal bar in gymnastics. In all of these cases, the auxiliary sub-action's goals aim to optimize the initial state of the respective main sub-action that, in turn, is directly related to the overall action goal.

**Figure 2:**
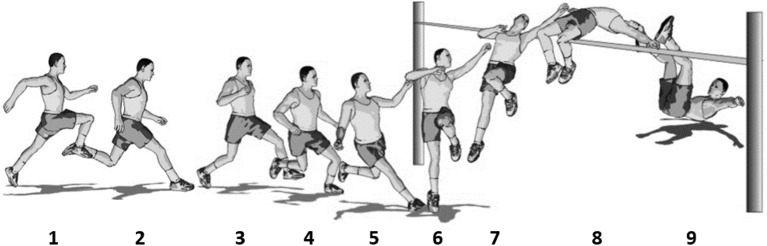
**An action sketch of the Fosbury flop (pictures 1–9) illustrating the sub-actions “straight run-up” (1–2), “curved approach” (3–5), “take-off” (5–6), “ascending” (7), “bar clearance” (8), and “landing” (9)**.

After having identified functionally defined (main and auxiliary) sub-actions, the second step of a functional movement analysis results in an “action sketch,” that means, a technique-related structure that embraces all crucial sub-actions. In Figure 2, such an action sketch is depicted using the high-jump technique of the Fosbury flop as an example. The figure comprises nine “snap shots” (1–9) of the motion sequence that illustrate the functionally identifiable sub-actions of “straight run-up” (1–2), “curved approach” (3–5), “take-off” (5–6), “ascending” (7), “bar clearance” (8), and “landing” (9).

### Modalities

From a functional point of view, it is not only decisive for an optimal performance to execute actions, and on a finer grain size, sub-actions; additionally, it is crucial to execute these actions and sub-actions in a certain way. In vaulting in gymnastics, for instance, the run-up should be performed in a constantly accelerating manner so that an optimal locomotion speed is reached just before the last step onto the springboard because, otherwise, the energy that could be invested into the jump gets lost. Another example would be the badminton smash that not only requires an arm movement against the shuttle that is accompanied by a pronation of the forearm in order to further increase the speed of the racket; instead, the forearm pronation needs to be executed explosively and at the last possible moment in time in order to avoid an increase of air resistance due to a forward-facing racket. Finally, when performing a B-line stall in paragliding, that means, a deliberate fast descent by pulling certain lines of the wing down and thereby deforming its shape, the lines not only need to be released at a certain point in time; instead, they need to be released symmetrically and carefully in order to prevent the wing from shooting forward, and even worse, from shooting forward asymmetrically.

In the action-oriented approach to functional movement analysis by Göhner (2013), those specifications of actions or sub-actions are labeled “modalities.” When exemplifying the concept of modalities by the Fosbury flop that has already been used in Figure 2 to illustrate the step of identifying sub-actions, one would have to demand, among other things, that the athlete accelerates over 5–7 steps of the straight run-up, inclines over the three steps of the curved approach, takes off with the leg farther away from the bar, lets the free leg drop down over the ascending phase, changes from an arched to a L-position for bar clearance, and finally, lands on the rounded back.

### Functional assignments

As it should have become apparent throughout the descriptions so far, the credo of a functional approach to movement analysis is based on the idea that action-related movements as well as modality-related specifications should not be understood as mere physical processes defined in space and time but should be approached from a perspective focussing on actions, sub-actions, and modalities that are determined by their function with regard to achieving certain goals or sub-goals. Consequently, it is less the observed movement that drives the assignment of functions but more the search for assignable functions that reversely drives the structuring of observed movements. Hence, when it comes to the identification of actions, sub-actions and modalities, the respective functional assignment must have already—and implicitly at least—been present as, otherwise, these steps simply could not have been taken. Nevertheless, as soon as actions, sub-actions, and action modalities have been successfully identified, based on, maybe implicit functional assignments, it makes a lot of sense to complete the functional analysis by making the respective assignments explicit. This means that at the end of a functional analysis, it must be possible to add an “in order to” clause to each and every action, sub-action and modality since, if such a specification could not be added, it seems doubtful whether the respective item can actually claim functionality with respect to the to-be-achieved goal at hand.

In Tables 1, 2, the reader was already provided with a number of examples of how those “in order to” statements should be expressed and how the respective function can be derived from different domains as from biomechanics, physiology, etc. In the following, the assignment step will be further illustrated by adding the required statements to the modalities which were already sketched above for the sub-actions of the Fosbury flop as depicted in Figure 2:

- The straight run-up (1–2) is composed of 5–7 straight steps with increasing speed *in order to* reach an individual optimum for being transformed into an optimal vertical take-off speed because sport practice shows that in this way, the optimum speed is achieved most easily and reliably.
- The curved approach (3–5) is accompanied by an incline away from the bar *in order to* produce a centrifugal force that will, after the termination of the incline with the last step, propel the athlete over the bar.
- The take-off (5–6) is accompanied by a diagonal swing of the free leg *in order to* produce a rotation of the whole body, and by this means, to prepare an optimal clearance position backwards over the bar.
- The ascending phase (7) is, at its end, accompanied by a drop of the free leg *in order to* achieve an optimal preparation of the subsequent clearance position.
- Over bar clearance (8), the arch position is changed to a L-position *in order to* maximally exploit the maximum height of the body's center of mass because in both positions, the current bar-clearing part of the body is, at the costs of other body parts that are actively pushed downwards, positioned as high as possible.
- As a matter of course, the landing position (9) does not affect the overall jumping performance anymore; however, a rounded back is important *in order to* prevent neck injuries.

With the completion of assigning functions to actions, sub-actions and modalities, the functional analysis of the movement at hand has been finalized. For an even deeper understanding of the procedure as well as for gaining a deeper insight of how a functional movement analysis could contribute to the identification of alternative task solutions, in Appendix A of Supplementary Material, a functional analysis of the golf swing is worked out in even more detail.

## From task analysis to motor control: Behavioral shaping, modular controllers, and explicit concepts

Before debating the question of how the adoption of a functional framework could support the practice of sports and physical education, it might be helpful to delineate the approach introduced here from related concepts that can be found in movement-science literature. In this respect, three theoretical links can be identified: a first one regarding the shaping of the observed behavior; a second one referring to the level of cognitive motor control; and a third one being related to the psychological level of explicit goal setting.

### Constraints and behavioral shaping

From a dynamical-system perspective, movements are not the product of rigid, centrally stored entities like “motor programs” (e.g., Schmidt, 1975). Instead, it is assumed that skilful behavior results from high-dimensional interactions within and between the actor and the environment. Following this idea, movements are conceptualized as a phenomenon of dynamical-system emergence. Consequently, the role of motor control is reduced to contributing to the self-organized “shaping” of the observable motor behavior over the course of action.

The affinity between a dynamical system approach to motor control and a functional movement analysis refers to the strict prioritization of the action goal over the movement itself. On the basis of such a prioritization, the observation does not come as a surprise that, for instance, expert blacksmiths are distinguished by a remarkable constancy of the hammer's working point trajectory that cannot be predicted from the rather variable kinematics of the joint angles. Hence, the goal-related measure shows less variance than movement-related measures—despite the fact that, in the end, the degree of goal achievement is entirely determined by the observable movement. This constancy phenomenon was empirically described first by Drill (1933), a member of the Klemm group of the Leipzig school of Gestalt psychology; it was termed “functional equivalence” by Bernstein (1947/1996) in his famous paper on “dexterity and its development.”

When the essence of behavioral invariance refers to the action goal that, in turn, can be achieved by a variety of functionally equivalent movements, the question arises: What kind of influences contribute to the process of the emergence of a particular movement? This question is answered by (Newell, 1989) with a taxonomy of action-relevant constraints (see also Newell and Jordan, 2007; for a review, Van der Kamp et al., 1996). In the original (Newell, 1989) scheme, constraints are classified into task constraints (e.g., instructions by the coach), environmental constraints (e.g., playground specifics), and organismic constraints (e.g., athlete's muscle strength). On the basis of such an understanding of motor control, Bril et al. (2012) suggest that for the development of motor expertise in the use of certain tools, functional parameters not depending on the actor (in hammering, e.g., the point of percussion) are increasingly concerted with control parameters that are under the control of the actor (in hammering, e.g., the velocity at impact). Movement parameters (in hammering, e.g., joint kinematics), in turn, are thought to arise from this coordination in consideration of regulatory parameters (in hammering, e.g., muscular effort) in order to shape an optimal movement variant within the space of functionally equivalent task solutions. On an empirical level, Parry et al. (2014) were able to show that tool-use ability actually seems to be guided by those functional dynamics and not be driven by an internal optimization process regarding the specification of kinetic joint profiles.

When comparing a dynamical system perspective with the here proposed functional approach to movement analysis, beyond the feature of emphasizing goals rather than movements, the idea that optimal task solutions are specified by constraints can be found in both theoretical frameworks (see Figure 1; in the movement-analysis approach originally labeled “ablaufrelevante Bezugsgrundlagen” by Göhner, 1979). More precisely, goal- and rule-related functional constraints seem to be closely related to Newell's task constraints, environment-, object-, and device-related functional constraints to Newell's environmental constraints, and athlete-related functional constraints to Newell's organismic constraints. Hence, when appraising the fundamental impetus of the two concepts, the basic idea that an entity is not given as such but arises from affecting variables is identical indeed.

At the same time, however, the two concepts differ in another important respect. This difference results from the respective focus of interest that lies on the emergence of observable movement patterns in dynamical-system theory and on the differentiation of to-be-achieved (sub-) goals in the case of Göhner (2013). More precisely, Göhner (2013) is mainly interested in the task to be solved, that means, in the first place, independent from an actor and his or her capabilities to execute an appropriate movement. This, as a matter of course, does not imply that Göhner (2013) denies the existence of an actor who, in the end, is responsible for solving the task—as, for principal reasons, a task cannot be thought of without an actor. Nevertheless, for Göhner (2013), the task as such, as it presents itself to all humans (or to a defined sub-group of humans at least), is of major interest in the interdependency of goal-relatedness and constraining factors. Hence, on the one hand, as explicated above, a functional approach to movement analysis seems to fit a dynamical-system framework of motor control quite well; on the other hand, the approach should be regarded as fundamentally neutral with respect to particular concepts for the explanation of human motor control and coordination—with respect to a dynamical-system perspective as well as to the theoretical concepts that will be discussed in the following.

### Internal models and modular controllers

As explained before, a functional approach to movement analysis can be much better married with a dynamical-system than with a motor-program framework as, from the former perspective, the action goal is prioritized over the movement whereas the opposite is true from the latter. However, the underlying controversy between action and motor perspectives on movement control (Meijer and Roth, 1988) can be treated in two distinctly different ways. On the one hand, the focus can be laid on the perspectives' differences regarding the role of emergence vs. prescription, thereby fanning the flames of the old controversy. On the other hand, from a cognitive-motor perspective, one could appreciate the fact that the need for internal representations—although not vanishing—is considerably reduced if a substantial part of the movement emerges from dynamical interactions. This would imply that the cognitive contribution to coordinated movement behavior is restricted to the fixation of goals and to the provision of the substrate that is needed to implement the crucial calculations for the generation of appropriate efferent commands.

In this respect, over recent decades, a remarkable change could be observed in the cognitive branch of movement science away from prescriptive models like Schmidt's *schema theory* (1975) toward conceptualizations of motor control that are fundamentally rooted in the belief that movements are controlled in terms of the anticipation of action effects. Empirically, this view could be supported by a multitude of studies, for instance, by Kunde (2001) who was able to show that in a two-choice reaction-time task responses of different intensity (forceful vs. soft press on a response key) are initiated faster if a compatible (forceful/loud, soft/quiet) rather than an incompatible (forceful/quiet, soft/loud) auditory effect is produced as a consequence of the key press. Thus, it could be demonstrated that the action effect was taken into account already during the generation phase of the movement. Obviously, this reorientation away from movement-related motor programs toward the anticipation of the action effect shifts the basic idea of cognitive motor control pronouncedly into the direction of goal-directedness that is definitive for a functional approach to movement analysis.

In current movement science, the idea of effect-related motor control is generally linked to the *ideo-motor principle* that is characterized by Kunde et al. (2004, p. 88) as “radical in the sense that it considers actions to be exhaustively represented in terms of their re-afferences, and that thus there is no other way to intentionally select and/or initiate an action than by anticipating its sensory effects.” The *ideo-motor principle* can be traced back to James (1981/1890) and its revitalization by Greenwald (1970), and more recently, by Prinz (1987) who was able to prove that due to mapping problems between otherwise isolated perception and action codes, movement planning *necessarily* happens in terms of perceivable action effects. Within the family of effect-related motor-control theories, the computational-cognitive concept of *internal models* has probably become the most widespread in international movement science (e.g., Wolpert et al., 1995; for adaptations to issues of sports: Hossner, 2004; Schiebl, 2008). In this concept, the effect anticipation is realized by an internal *predictor*, a so-called *forward model*, and the generation of movement commands is carried out by an internal *controller*, a so-called *inverse model*. The controller is assumed to be structured over the learning process with respect to the effect expectations that can be derived from the predictor. Translated into the language of a functional approach to movement analysis, this would imply that an action or sub-action refers to an internally perceivable state that in turn, can be equated with the output of the internal predictor and the input of the controller, respectively. Consequently, movements would be controlled in terms of the anticipation of their effects.

Regarding details of the controller's work, two variants of the internal-model concept can be distinguished, a holistic one based on a single predictor-controller combination that would conceptually be responsible for the totality of motor control processes, and a modular one with multiple pairs of models that are thought to be responsible for the achievement of certain aspects of the task at hand (Wolpert and Kawato, 1998). According to Fodor (1983), those modular sub-structures would be exploited best if they operate in an “informational encapsulated” manner. Empirically, Thoroughman and Shadmehr (2000) were able to show that grasping movements—that is, movements that are determined by a single goal—are produced by the adaptive combination of “motor primitives” that, in the context at hand, can be understood as functional modules. From a functional perspective on movement analysis, such a modular concept is particularly appealing when the achievement of a goal or sub-goal requires the combination of movement elements that can be functionally separated from each other. When performing a Fosbury flop, for instance, the take-off requires (a) a forceful knee extension in the jump leg that is accompanied (b) by a diagonal swing of the free leg as well as (c) by an optimally coordinated arm swing. Over the course of learning, an athlete might perceive these elements as fundamental “building blocks” for the successful achievement of the sub-goal “take-off” (Hossner, 1995). Consequently, it is plausible to expect that the sub-goal-related micro-structure derived from a functional task analysis is directly reflected by the modular architecture that is acquired by the athlete on the level of motor control. Of course, such a one-to-one match does not necessarily need to be developed; however, if one is willing to accept the matching hypothesis, a functional movement analysis would provide a fruitful starting point for the identification of modular components that actually *are* responsible for the control of specific aspects of complex motor behavior.

### Action control and explicit concepts

One important point should be added to the discussion of the relation between functional movement analysis and motor-control architectures. This point refers to the fact that the modular structures discussed so far are fundamentally neutral regarding the distinction between explicit and implicit processes. Hence, the idea of a cognitive control architecture with modules that reflect the world as it is functionally subdivided by the behavioral accessibility by a human agent is thought as working independently of any kind of explicit awareness about what happens within the modules (for perceptual modules, see Fodor, 1983). Thus, the internal motor control architecture would refer to knowledge that perfectly fits Magill's (1998) statement that “knowledge is more than we can talk about.”

Nevertheless, it should also be admitted that there is also movement-related knowledge we definitely *can* talk about, that is, knowledge that, in the context at hand, refers to the explicit representation of actions, sub-actions and modalities as well as to functions that can be assigned to these entities. In balancing tasks, for example, it is probably completely impossible to consciously access how several functional sub-systems operate and interact in order to prevent the actor from tumbling from the balance beam. However, as it is well known from sports practice, balancing performance can be improved quite easily and effectively when pursuing the explicit strategy of anchoring the gaze on a distinguished point at the wall. On the one hand, this improvement can be ascribed to the work and interaction of certain modular control structures that actually produce a balanced body position; on the other hand, without any doubt, the anchoring of the gaze can be achieved by a process that is driven by conscious awareness or that is accompanied by conscious awareness at least. Hence, it may also be of interest to identify explicit processes that can be regarded as correlates of motor control structures that, in turn, may reflect the functionality of the behaviorally accessible world as it can be structured by a functional movement analysis.

In this regard, such a structural model on the level of action control has been proposed by Schack (2004, 2010). This model is based on the identification of so-called “basic action concepts” that are understood as “cognitive compilations of movement elements and body postures that share functions in the attainment of action goals” (Schack, 2012, P. 205). Methodologically, these basic action concepts are derived from the application of a method called “structural dimensional analysis of mental representation” that is based on a distance scaling between selected representational concepts, a subsequent structure analysis by a hierarchical cluster analysis, furthermore, a dimension analysis of the established representation, and finally, an invariance analysis of the cluster solutions revealed (for details, see Schack, 2004). As the initial step of this procedure is based on ratings given by the athletes regarding the subjectively perceived functional proximity between pairwise combinations of action concepts, we would like to refer to the finally derived mental representations as explicit representations, not necessarily in the sense that the derived structure can be explicitly recalled and reproduced but that the method aims at consciously assessable knowledge as, otherwise, a proximity rating would be infeasible. Schack (2004, p. 408) himself assigns the resulting hierarchical structure of basic action concepts to a level of mental representation (III) that is below a level of mental control (IV) but above a level of sensorimotor representation (II) and a level of sensorimotor control (I). With respect to hypothetical interdependencies between these levels, Heinen (2005) was able to empirically show for artistic-gymnastic routines as well as for volleyball skills that the structure of mental representations derived by this means actually correlates with certain kinematic features of the observable movements.

Thus, from the perspective developed here as well as on the basis of the representational concept suggested by Schack (2004), “one important step for further research in movement science could be to perform a systematic search for paths between biomechanical aspects and functional units of movement organization” (Schack, 2004, P. 428). As we think, the functional approach to movement analysis seems to be a perfect candidate to satisfy this demand. Hence, it does not really come as a surprise that the respective link to Göhner (1979) has been made by members of the Schack group themselves (e.g., Heinen, 2005; Bläsing and Schack, 2012). In our eyes, the relation between the levels discussed here would be a rather indirect one being based on (a) a concertation of functionally derived task demands with, on the actor's side, the structure of the control-parameter space, (b) an actor-internal modularisation of this parameter space in accordance to separable functions that subserve the achievement of a sub-goal or the overall task goal, and (c) an explicit re-description of this structure on the level of explicit mental representations.

As already stated previously for the level of dynamical-system emergence as well as for the level of effect-related modular control, it need not necessarily be true that an explicit structure in terms of hierarchically organized basic action concepts meets the structures that are assumed on a motor-control level or that are derived by means of a functional movement analysis. However, as long as basic action concepts are sufficiently distinguished from the sub-actions resulting from a functional movement analysis, a functional approach might also serve as a valuable heuristic for the identification of explicit mental representations and vice versa.

## Functional thinking in sports: Part practice, functional equivalence, technique innovations, personal style, and root causes of movement errors

For the methodology of sports and physical education, first, Göhner (2013) emphasizes the usefulness of a functional movement analysis when it comes to the segmentation of movements for the purpose of facilitating complex motor-skill learning. In this regard, it can be inferred that movements should be split up if and only if the resulting parts make sense from a functional point of view, that is, if and only if they reflect certain functions that, in turn, are connected to certain sub-goals. Furthermore, when pursuing a part method, the question arises as to whether the initial functional state for a certain movement part can be guaranteed to a sufficient degree. If this is not the case, the teacher or coach has to take care that, due to the isolation, the missing previous function is adequately replaced. Finally, when isolating movement parts, the learner might be confronted with the acquisition of units that refer to auxiliary sub-actions implying that the learner would supposedly aim at achieving a sub-goal that might subjectively not correspond to the overall movement goal. To circumvent this problem, Göhner (1975a,b) suggests refraining from practicing auxiliary sub-actions in isolation but to start with the sub-action that is directly related to the ultimate movement goal. Consequently, auxiliary sub-actions would have to be replaced initially by making use of appropriate means (in gymnastics, for instance, physical guidance by the teacher) that, over learning, could be removed gradually. Thus, the overall movement goal would guide the learner from the very beginning of the methodologically ordered series of exercises and would be present over the whole course of learning.

Beyond these implications for the organization of part practice, a functional approach to movement analysis seems to be of particular value for the practitioner when he or she is expected to provide the learner with augmented feedback because errors, as explicated in the introduction to this paper, can be defined as deviations of the actual from the desired technique value. If this desired value is determined on the basis of a functional analysis, the teacher's or coach's attention is not directed anymore to the question of whether the observed movement deviates from movements that are produced by top-level athletes; instead, the focus is laid on the question of whether functionally defined demands are met by the observed movement or not.

The consequences of this reorientation away from spatio-temporally defined “ideal” movements toward to-be-fulfilled functions may be quite drastic. In basketball, for example, a particular throwing technique can claim superiority over others in which the elbow points exactly into the direction of the hoop. The functional reason for this superiority is connected to the fact that in the one-handed overhand throw, the movement must be controlled in the plane of the resulting ball curve only. Whether this reduction of degrees of freedom is realized, from either an initial position with the ball over the head or an initial position with the ball in front of the trunk is functionally of minor importance. Hence, although the second-mentioned technique is less common in top-level basketball, this variant should not be regarded as an error. Instead, the existence of two functionally equivalent task solutions should be conceded.

The number of optimal task solutions is typically further enlarged when environment- and athlete-related constraints are taken into account. In this respect, it can be stated that the one-handed throwing technique becomes non-functional as soon as the distance between the player and the basket exceeds a certain strength-related threshold. For this reason, even in NBA basketball, two-handed shooting movements can be observed, in particular, when ball possession changes after a defensive rebound and only a second playing time remains. In those cases, the player is forced to shoot over a very long distance whilst, for average players at least, the muscular strength that can be invested in a standard throwing technique does not suffice to produce a ball speed that is needed to cover the whole distance. The very same issue would arise if it is not the top-level player who is overcharged by the long-distance throw but a young child who is positioned close to basket but whose arm strength, nevertheless, does not suffice to successfully perform a classical one-handed throw. In those cases, once again, a two-handed throw—or an underarm throw that would allow ball acceleration even better—should not be regarded as an error but as the functionally optimal task solution.

This kind of functional thinking seems of particular value also for technique training in top-level sports that is characterized by the problem that only a small number of athletes exist that could be taken as a role model. Consequently, in elite sports, a functional analysis should be even less rooted in observable movements but in functions that come along with the to-be-fulfilled task. A good example for the pursuit of exactly such an approach is the technique evolution in top-level ski jumping. As it seems functionally appropriate to support the ski-jump take-off by swinging both arms upwards, in the first half of the twentieth century, exactly such an arm swing was produced by the best athletes leading to a flight position with both arms extended forward. A thorough functional analysis, however, would have to include the fact that the jumped distance also depends on aerodynamic efficiency that, on the contrary, would be optimized by holding the arms backwards close to the body. The fact that today's ski jumpers prefer the second-sketched posture, thereby completely turning down the advantages of an upward arm swing, points to the fact that the optimization of aerodynamics is biomechanically more important than the take-off support by arm movements. Hence, technique-related innovations in top-level sports seem to require coaches who dedicate themselves to an in-depth understanding of the functional structure of the to-be-optimized movements.

A further consequence of a functional approach in the context of error identification refers to the fact that parts of the movement that allow for functional assignments can be distinguished from other parts for that such an assignment seems impossible. In those cases, in the first instance, errors can only be identified with respect to functionally determined sub-actions. With respect to movement parts without a functional assignment, two categories should be distinguished. The first category refers to parts that are completely dispensable from a functional viewpoint. This applies, for instance, to a spacious preparation of the pole plant in deep-powder skiing. Whenever, dispensable movement parts are observed by the teacher or coach, these parts should also be regarded as an error as, in the ski example, over-spacious arm movements increase the risk of losing balance and are thus a potential cause for subsequent faults. However, dispensable movements should be regarded as errors in a weak sense with the consequence that these details should not attract the teacher's or coach's main attention. The second category of parts without a functional assignment refers to movements or postures of body limbs that are not involved in the achievement of current goals. In foil fencing, for example, that is defined by the reduction of the target area to the torso, the free arm is of definite importance to keep balance; however, as long as this sub-goal is achieved, a huge variety of different arm and hand postures would perfectly fulfill this function. In those cases, the teacher or coach should not correct the athlete's individual solution but treat his or her specific posture as a matter of personal style.

Finally, in the context of error identification in physical education and sports, a functional movement analysis pinpoints the fact that observable movements typically result from the realization of a number of interacting sub-actions that are interwoven in a complex fashion. As detailed previously, these sub-actions are defined by initial and final states. As final states of previous sub-actions regularly determine initial states of subsequent sub-actions, it can be concluded that errors that are observed at some point in the movement need not be ascribed to the currently executed sub-action; instead, the root cause might be found in a previous sub-action. As a matter of course, if the detected error is expected to be a subsequent error, the learner should be provided with augmented feedback regarding the actual error cause. On the side of the teacher, however, the fulfillment of this demand requires a well-developed understanding of the functional interrelationships in the motor task at hand.

Summing up, in sports practice, a functional approach to movement analysis helps:

- to identify the existence of more than one task solution that can claim functional equivalence;
- to distinguish functionally equivalent task solutions from variants that are superior with respect to specific environment- or athlete-related constraints;
- to gather, especially in elite sports, the competence to deal with the problem of desired technique values in an innovative fashion;
- to focus the teacher's or coach's attention on the parts of the movement that are crucial for achieving the overall movement goal;
- to differentiate between movement parts with and without a functional assignment;
- to provide the learner with corrective feedback on movement parts without a functional assignment only in cases of overall detrimental consequences;
- to treat movement parts without a functional assignment and without a high probability of overall detrimental effects as personal style that can be left uncorrected; and
- to trace back an observed error to its root cause and to give feedback on the actual rather than on the subsequent error.

Hence, it seems worthwhile to disseminate a functional framework of movement analysis in the world of sports practice and to make functional thinking accessible to teachers and coaches on each level of performance from teaching beginners in physical education up to conducting technique-training sessions in top-level sports.

### Conflict of interest statement

The authors declare that the research was conducted in the absence of any commercial or financial relationships that could be construed as a potential conflict of interest.
