# Supplementary material for: A functional approach to movement analysis and error identification in sports and physical education
Source: Front Psychol. 2015 Sep 10;6:1339. doi: 10.3389/fpsyg.2015.01339 (PMC4564696; doi:10.3389/fpsyg.2015.01339)
Supplement: Supplementary file 1 [file AppendixA.PDF]

# A functional approach to movement analysis and error identification in sports and physical education

**Ernst-Joachim Hossner[1], Frank Schiebl[2] and Ulrich Göhner[2]**
[1]Institut für Sportwissenschaft, Universität Bern, Bern, Switzerland
[2]Institut für Sportwissenschaft, Eberhard-Karls-Universität Tübingen, Tübingen, Germany

## Appendix A.
## An applied example: The free-release golf swing as a functionally optimal task solution

For the purpose of gathering an in-depth understanding of the functional approach to movement analysis, in the following, a detailed analysis of a particular sport task, the analysis of the golf swing, will be presented. Due to the core target of golf to move the ball into a hole by a minimum of shots, the golf swing aims on hitting the golf ball precisely with a defined movement speed at impact, and regarding spatio-temporal features, as reproducible as possible. From a functional point of view, the desired movement should be reduced to the essentials, that is, to those details that are functionally required in order to optimally fulfil the task. The *free-release method* proposed by Drollinger (2012) aims exactly on such a reduction to the essentials.

When beginning the functional analysis with a review on views held in sports practice and on scientific findings, the review shows that assumptions on the core structure have not changed over the last decades (e.g., Cochran & Stobbs, 1969; Hogan & Wind, 1985; Hume, Keogh & Reid, 2005). Accordingly, the golf swing can be split into the movement phases of setup, back swing, down swing, impact, follow-through and finish. As the impact is entirely determined by the down swing and the finish by the follow-through, four crucial sub-actions should be distinguished from a functional perspective: setup, back swing, down swing, and finish. These sub-actions can be functionally further sub-divided following a finer grained level of analysis (for details, see Figure A1).

When having a closer look onto the sub-actions' microstructures, substantial differences can be revealed in literature. This is particularly true for the functional assignments regarding modalities. In this respect, whilst there is broad consensus on the action's overall goal, the question of how this goal is optimally achieved is typically reduced to the discussion of energetic aspects, especially regarding the acceleration of the club head (e.g., Brown, Selbie & Wallace, 2013; Cheetham, Martin, Mottram & St. Laurent, 2000; Chu, Sell & Lephart, 2010; Nesbit & McGinnis, 2009; Sato, Kenny & Dale, 2013; Torres-Ronda, Sánchez-Medina & González-Badillo, 2011). In contrast, the fact that the movement also has to meet precision requirements is underrepresented in this discussion, although it is generally accepted that the golf swing should be regarded as an optimisation problem in the first place (e.g., Hetu, Christie & Faigenbaum, 1998; Hume et al, 2005). Hence, with a few exceptions (Ball & Best, 2007; Drollinger, 2012; Peper & Tiegreen, 2005; Snead & Stump, 1962), ideas are missing on how an athlete can improve accuracy. This is not only surprising from a performance-enhancement point of view but even more from a prevention perspective because statistics clearly testify that trials not performed in a precise manner are the number one reason for golf injuries (Batt,

1992; McHardy, Pollard & Luo, 2006). At this point, a functional perspective comes into play, according to this approach, as precision also demands and the prevention from overload must be considered when it comes to the definition of an optimal golf swing technique. Hence, these demands will be considered in the following. For the sake of simplicity, the following descriptions and analyses were reduced to right-handed players.

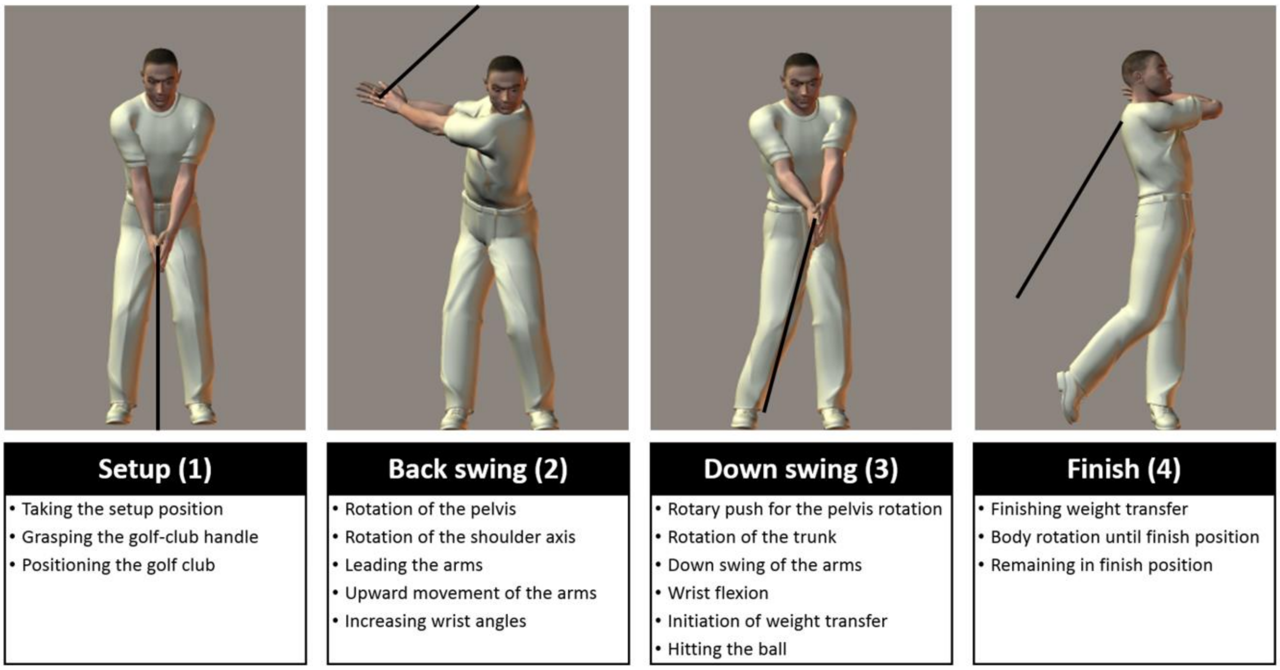


*Figure A1. Sub-actions of the golf swing according to the free-release method*

## Sub-action "setup"

The setup aims on the provision of a body posture that can claim optimality with respect to the execution of subsequent movement parts. On a micro-level, it is composed of the sub-actions of taking the setup position, grasping the golf-club handle, and positioning the golf club in relation to the golf ball (for details, see Figure A2 and Table A1).

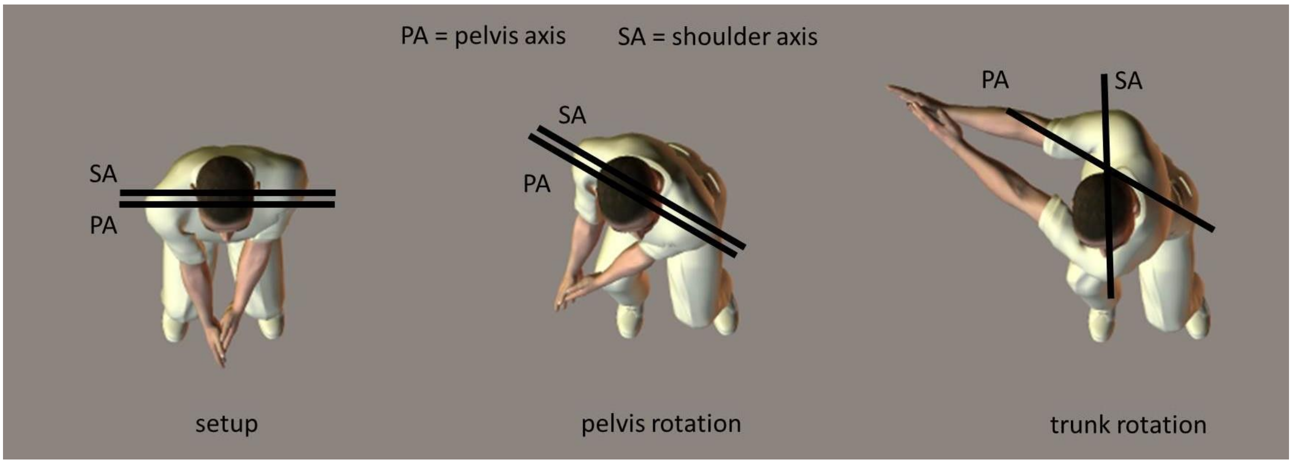


*Figure A2. Transversal view of the setup and back swing of the golf swing according to the free-release method*

*Table A1. Functional Assignments for Modalities of the Golf Swing Sub-action "Setup"*

| | Modalities | Functional Assignments |
|---|---|---|
| **a** | **Taking the setup position** | |
| a1 | Weight distribution is on both feet | In order to ensure the highest possible postural stability |
| a2 | The axes of the shoulder and of the pelvis are aligned parallel to the target point | In order to attain an optimal orientation in the direction of the target |
| a3 | The feet are not misaligned and the length axes of the feet are parallel | In order to attain an optimal acceleration of the club head and to attain an optimal hit position at the down swing |
| a4 | The trunk is positioned with a non-bended spine with respect to the vertical with an angle of approximately 35°-40° | In order to allow, from the very beginning of the movement, a rotation around the length axis of the trunk without a change of the trunk's vertical angle and in order to attain an exactly reproducible final position of the back swing |
| a5 | Both legs are bent within a knee joint of approximately 25°-30° | In order to be able to perform an optimal, low stressing rotation of the pelvis over the back and down swing because an optimal mobility of the pelvis is only given if the knees are in a flexed position because in this case, the second order function regarding the rotation of the lower leg can be used |
| a6 | Stance is a little wider than pelvis width | In order to allow an optimal active movement of the right leg and a rotation of the pelvis in the direction of the target point and in order to allow a controlled weight transfer to the left foot over the follow through without the necessity to perform a push with the right leg for reaching the finish position |
| **b** | **Grasping the golf-club handle** | |
| b1 | The handle is grasped with an overlapping grip | In order to get a powerful grip on the golf club to be able to control the golf club accurately |
| **c** | **Positioning the golf club** | |
| c1 | The distance from the thigh to the top of the golf club matches approximately the size of the palm | In order to force the setup of the specific body-joint angles and in order to force a close contact between the trunk and the upper arm which increases the amount of tactile information which, in turn, can be used for an exact striking of the setup position |
| c2 | The direction of the club's length axis is perpendicular to the target direction resp. to the pelvis axis | In order to achieve a neutral setup position of the impact plane for avoiding hooks and slices |
| c3 | The club position is always in the middle between the left foot and the right foot | In order to ensure that the player does not need to change the swing kinematics when using different golf clubs |

Taking the setup position can claim functionality only if the feet's length axes are aligned in parallel as only in this case an optimal turning mobility of the pelvis is given because the adductors of the lower left extremities are not in a pre-stretched condition. An optimal turning mobility is required in order to achieve a back-swing finish position that enables the player to generate a maximum acceleration of the body segments and of the club. However, note also that this alignment leads to a high axial stress on all joints of the left lower extremity, particularly in the later achieved finish position. One possibility for reducing this stress would be to wear special shoes that allow a turn of the left foot after ball impact. With respect to the width of the stance, a stance considerably wider than the length of the pelvis transversal axis leads to a greater support plane of the athlete's centre of mass. However, such a wide stance also results in a loss of centre of mass's height so that during the follow-through movement, the centre of mass must be elevated what, in turn, could be a source for errors in controlling the swing path. Hence, a reasonable compromise must be reached regarding the width of the stance. Furthermore, it can be stated that an optimal mobility of

the pelvis is only given if the knees are in a flexed position because only then the second-order function regarding the inside and outside rotation of the lower leg can be used.

## Sub-action “back swing”

The back swing aims at the achievement of an optimal body position for the subsequent down swing. On a micro-level, it covers a clockwise rotation of the pelvis, a clockwise rotation of the shoulder axis, an arm movement that can be further split into leading and upward phases, and an increase of the wrist angles (for details, see Figure A3 and Table A2).

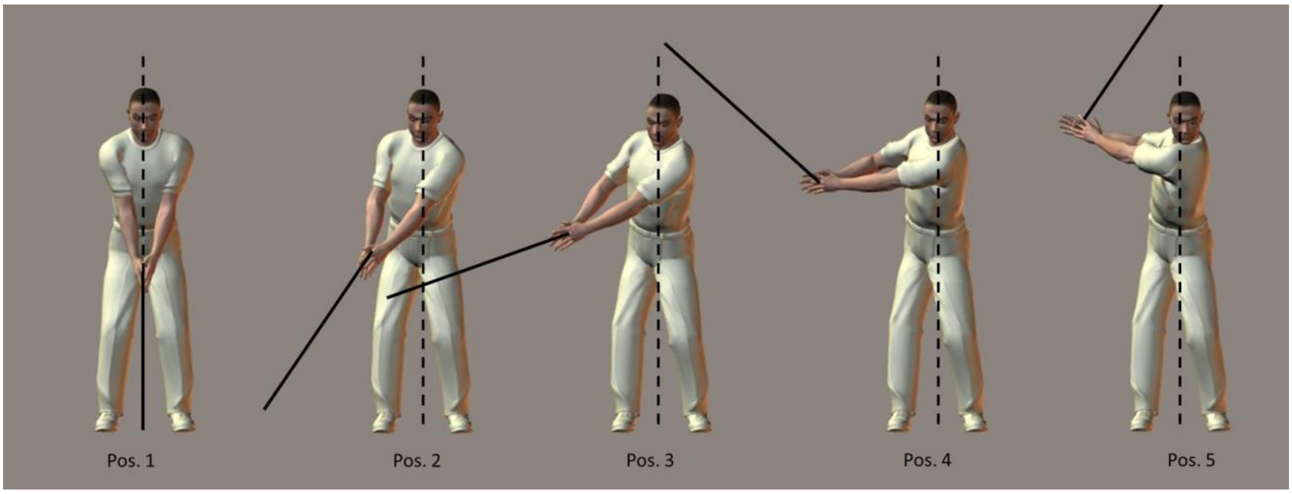


*Figure A3. Frontal view of the setup and back swing of the golf swing, according to the free-release method, without lateral weight transfer*

From a functional perspective, a complete weight transfer over the back swing onto the right foot would make a push for the pelvis rotation at the beginning of the down swing impossible such that, in this case, the centre of mass could be accelerated in the vertical dimension only. The reason for this issue is that the force vector of the right leg’s push cannot work eccentrically with respect to the centre of mass because the body’s centre of mass is located perpendicular to the support plane. Hence, a turning push would only be possible after a shift of the centre of mass onto the left foot, which could be realised by several sub-actions. This shift, however, would necessarily come along with an increased risk of a volatile swing plane over the down swing. Consequently, an extensive weight transfer to the right leg would have a strong negative impact on the kinetic link chain which requires the turn of the pelvis as the first sub-action in the kinetic sequence (Kenny, McCloy, Wallace & Otto, 2008). For this reason, in the free-release method, a lateral weight transfer onto the right foot over the back swing is avoided.

Beyond demands on weight shift, over the back swing, the maximum rotation of the pelvis should not exceed 40-45° in order to avoid an overuse of the hip joints (McHardy et al., 2006). Furthermore, the maximum amount of shoulder rotation (cp. Figure A2) is constrained by the need to keep the ball in sight, which in turn, would become complicated by a shoulder rotation larger than 90°. However, as shown by Cheetham et al. (2000), an increased “X factor”, that is, the twist between the pelvis and the shoulder axis, does not automatically result in a higher acceleration. Overall, the rotation movements of the back swing are executed rather slowly (approximately 1.5 s) because it is well known from the speed-accuracy trade-off phenomenon that the higher movement speed is, the less possible it is to execute the movement with high precision (Fitts, 1954; Plamondon & Alimi, 1997).

*Table A2. Functional Assignments for Modalities of the Golf Swing Sub-action "Back swing"*

| | Modalities | Functional Assignments |
|---|---|---|
| **a** | **Rotation of the pelvis** | |
| a1 | The rotation is performed with a moderate speed around an axis between both feet | In order to avoid a weight transfer to the right leg which increases the risk of losing balance and in order to move the golf club directly to the desired horizontal position without an opening of the arm-trunk angle |
| a2 | The maximum rotation of the pelvis does not exceed approximately 40°-45° | In order to attain an optimal (where optimal means that the player is still able to see the ball, that as long as possible, acceleration is given ) position for the acceleration of the club over the subsequent down swing and in order to avoid an overuse of the hip joints |
| a3 | The decline of the pelvis axis with respect to the horizontal plane remains constant | In order to attain an exactly reproducible final position of the back swing and in order to minimise an undesirable impact on the swing plane over the down swing |
| a4 | Both knee joints remain flexed and both feet keep contact to the ground | In order to be able to keep the decline of the pelvis axis with respect to the horizontal plane constant and in order to allow an effective turn push of the legs over the down swing |
| **b** | **Rotation of the shoulder axis** | |
| b1 | A flexed spine as well as the decline of the trunk's longitudinal axis with respect to the vertical plane are maintained | In order to avoid an overuse of the vertebral joints and in order to attain an exactly reproducible final position of the back swing |
| b2 | The maximum rotation of the shoulder axis does not exceed 90° | In order to attain an optimal position for the acceleration over the down swing |
| b3 | The turn is performed with a moderate speed | In order to avoid an overuse of the vertebral joints and in order to attain an exactly reproducible final position of the back swing |
| **c** | **Leading the arms** | |
| c1 | Both arms, together with the club, form the shape of a "Y" | In order to achieve an optimal power and position control |
| c2 | The back swing is performed simultaneously with the rotatory actions of the pelvis and the spine | In order to keep the angles in the shoulder joints constant, and as a consequence, to reduce the system's degrees of freedom |
| c3 | Both arms keep contact to the trunk without changing the angles of the shoulder joints | In order to receive maximum tactile feedback which can be used for movement control and in order to reduce the system's degrees of freedom |
| c4 | The arms are moved as a consequence of the rotatory actions of the pelvis and the spine | In order to receive maximum tactile feedback which can be used for movement control and in order to reduce the system's degrees of freedom |
| **d** | **Upward movement of the arms** | |
| d1 | The back swing does not begin until the leading of the arms has been finalised | In order to use the tactile feedback of the arm-trunk contact as long as possible for the purpose of exact movement control |
| d2 | The final position of the back swing is achieved with a moderate movement speed | In order to attain an optimally reproducible acceleration position for the down swing and in order to keep the centre of mass balanced |
| d3 | The left arm does not rotate about its longitudinal axis | In order to avoid unnecessary movements |
| d4 | The left arm remains stretched | In order to achieve a reproducible final position of the back swing and In order to reduce the system's degrees of freedom |
| **e** | **Increasing wrist angles** | |
| e1 | The flexing starts as soon as the wrists pass the right thigh | In order to be able to optimally use all joints of the kinetic link for club acceleration over the down swing ("whiplash effect") |

Finally, regarding coordination, it is important that over leading the arms, no active movements are generated in the shoulder joints. Instead, the arm movement comes as a mere consequence of the pelvis rotation (cp. Figure A2 and A3: position 1-2). As research on intrinsic feedback indicates that augmented tactile feedback leads to a more precise movement control (Sigrist, Rauter, Riener & Wolf, 2013), in the functionally based free-release method, the use of tactile feedback over the back-swing phase is maximised. Furthermore, repeatability can be increased through freezing of degrees of freedom because through this, errors become less likely to occur (Beak et al., 2013). If, in contrast, the back swing is initiated by movements of the arms, as it is the case in other golf-swing techniques, the arms have to be moved in a lateral direction, which in turn, complicates balance control because of the resulting shift of the centre of mass.
As the free-release method is distinguished by the purity of its functional derivation, meaning that only those aspects of the swing are included that can be justified by functional means, it should not come as a surprise that the resulting movement differs from the technique classically taught in golf methodology (cf. Hogan, 1957; see Figure A4). In this respect, in the classical but not in the free-release golf swing, the weight is shifted to the right foot over the back swing resulting in a symmetrical weight distribution on both feet at the back swing's final position in the free-release but in an asymmetrical distribution in the classical variant (see Figure A3: positions 4-5 vs. Figure A4: positions 4'-5'). The particular disadvantage of the classical weight transfer to the right foot refers to the fact that, as a consequence, a subsequent rotary push of the pelvis is not possible so that the down swing must be initiated with either an arm swing or with a rotary trunk movement, which, in turn, would not be in accord with the segmental-interaction principle, which describes the most effective way of accelerating distal body segments (Hume et al., 2005; Knudson, 2007). Furthermore, if the down swing movement starts with a rotary action of the trunk, X-factor stretch is no longer possible. Hence, the functional approach of the free-release method offers a superior solution to the golf task at hand.

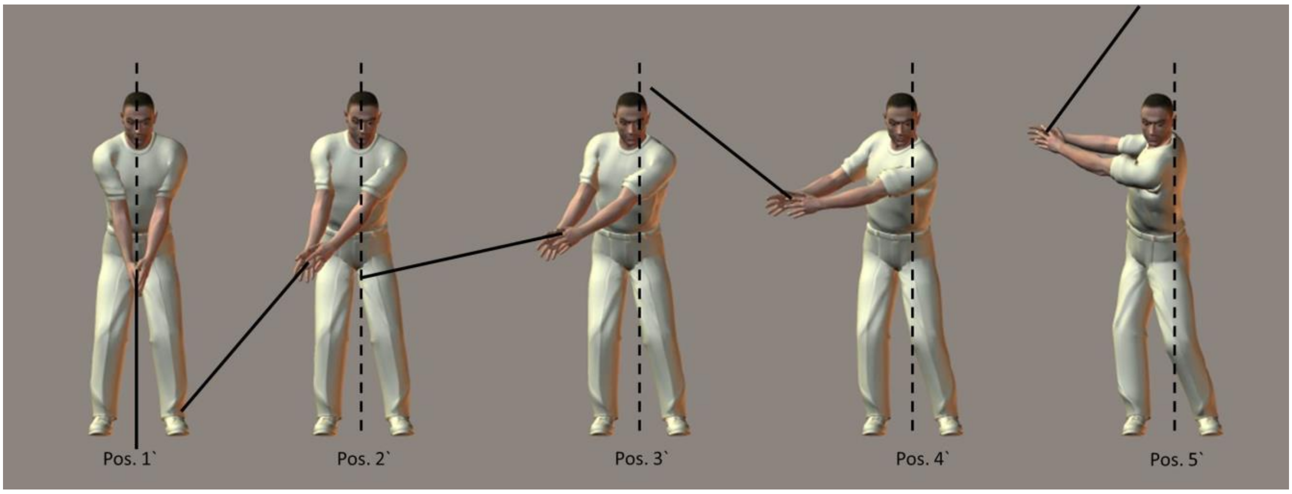


*Figure A4. Frontal view of the setup and back swing of the golf swing, according to the classical style, with lateral weight transfer*

## Sub-action "down swing"

The down swing aims at a precise hit of the ball in conjunction with the production of a pre-defined movement speed. Apart from this, the movement should be executed in such a way that a high repeatability can be guaranteed. The sub-action of the down swing can be further sub-divided into the rotary push for the pelvis rotation, the rotation of the trunk, the down swing of the arms, the wrist flexion, the initiation of the weight transfer to the left foot, and finally, the hitting of the ball (for details, see Figure A5 and Table A3).

*Table A3. Functional Assignments for Modalities of the Golf Swing Sub-action "Down swing"*

| | Modalities | Functional Assignments |
|---|---|---|
| **a** | **Rotary push for the pelvis rotation** | |
| a1 | The pelvis rotates in the direction of the target point without lifting the centre of mass | In order to be able to introduce maximum acceleration energy by the application of strong leg muscles into the movement system and in order to avoid an impact on the swing plane due to a change of the height of the centre of mass and in order to induce a pre-stretch of the muscles for optimising acceleration energy from the short-stretch cycle ("X-factor stretch") |
| a2 | The down swing is initiated by a movement of both legs | In order to keep the centre of mass stable because a translational movement could affect the desired swing plane negatively |
| a3 | Over the rotation movement, the centre of mass remains stable | In order to keep the balance on both feet, and as a consequence, have maximum control of the swing level |
| **b** | **Rotation of the trunk** | |
| b1 | The rotation is executed in the direction of the target | In order to be able to reach a maximum acceleration of the club head |
| b2 | The rotation is started immediately after the rotary push | In order to be able to reach a maximum acceleration of the club head |
| b3 | The rotation is executed without interruption up to the finish position | In order to be able to reach a maximum acceleration of the club head |
| **c** | **Down swing of the arms** | |
| c1 | The arms remain close to the body | In order to be able to hit the ball precisely and in order to avoid a volatile swing plane |
| c2 | The down swing of the arms starts as soon as the shoulder rotation has reached its maximum speed | In order to be able to reach a maximum acceleration of the club head ("whiplash effect") |
| **d** | **Wrist flexion** | |
| d1 | The wrist flexion is executed as soon as the arms are concentric in front of the body | In order to be able to reach a maximum acceleration of the club head ("whiplash effect") |
| d2 | The wrist flexion is executed rather passively by the active centrifugal force | In order to be able to reach a maximum acceleration of the club head ("whiplash effect") |
| **e** | **Initiation of weight transfer** | |
| e1 | The weight transfer to the left foot is initiated shortly before ball impact | In order to be able to link up the movement without interruption in the finish position |
| e2 | The plane of the pelvis does not change | In order to avoid an impact on the swing plane |
| **f** | **Hitting the ball** | |
| f1 | The face angle has (ideally) a value of 0° | In order to be able to realise a very accurate ball flight curve |
| f2 | The shoulder axis lies in parallel with the target direction and the pelvis axis is turned around approximately 20° farther than the shoulder axis | In order to be able to realise a very accurate ball flight curve |

From a functional point of view, the player has to act with both legs to keep the rotary axis stable. This means that the right leg pushes the right part of the pelvis into a left-forward direction and the left leg pushes the left part of the pelvis into a right-backward direction. When initiating the pelvis rotation, the trunk remains in the back swing final position for a short moment. Due to this behaviour, the so-called X-factor stretch

appears. Immediately after the start of the pelvis rotation, the rotation of the trunk follows whilst the down swing of the arms is performed simultaneously. In order to guarantee an optimal balance control, weight should be transferred to the left foot not earlier than just before the club hits the ball. A further reason for a late weight shift regards the avoidance of an interruption of the down swing, thus, allowing for a seamless transfer of the body segments to the finish position after the ball has been hit. The angle kinematics of the wrist joints over the first part of the down swing is characterised by an increased flexion of the right wrist and an increased extension of the left wrist, respectively. This movement surfaces as a rather passive action only if the wrists' angles are not fixed because the previously described change in joint angles is generated by the inertia of the club. Schneider (1990, p. 24-25) describes the principles of the intersegmental dynamics with interacting joint moments as a result of inertia and centripetal forces. Hence, the typical wrist-angle movement results in a whiplash-like down swing with the highest club-head speed ideally at the moment of impact.

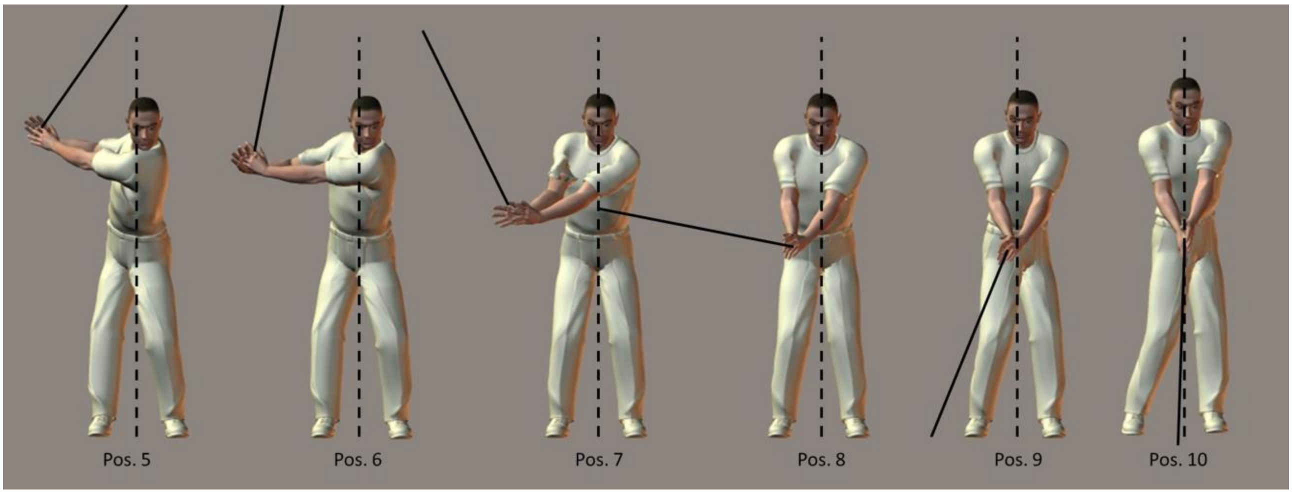


*Figure A5. Frontal view of the down swing of the golf swing according to the free-release method*

## Sub-action "finish"

The finish phase of the golf swing aims on finalising the movement in a smooth fashion. More precisely, a particular function of the finish regards the amortisation of the club's kinetic energy in a way that prevents bodily structures from overload. Micro-structurally, the phase covers the finishing of the weight transfer to the left foot, the rotation of the body until the finish position is reached, and remaining in this finish position for a certain amount of time (for details, see Figure A6 and Table A4).

For the finish position, a correlation between the final body position and lower-back injuries could be revealed (Sugaya, Tschiya, Moriya, Morgan & Banks, 1998; see also McHardy et al., 2006). For this reason, an appropriate execution of the finish phase is of major importance with respect to the prophylaxis of injuries. This function can be sufficiently fulfilled by a variety of final postures whereby details of the position seem to be correlated with different swing behaviours. However, it can be generally stated that the amortisation of the club's kinetic energy requires a high muscular effort, especially of the trunk muscles, in order to protect the spine against overload. This requirement can be met in a functional fashion by rotating the pelvis approximately 90° into target direction and moving the arms above the left shoulder such that the club targets diagonal to the ground behind the back. By this means, a hyperextension of the spine would be avoided (see Figure A6, Positions 10-12).

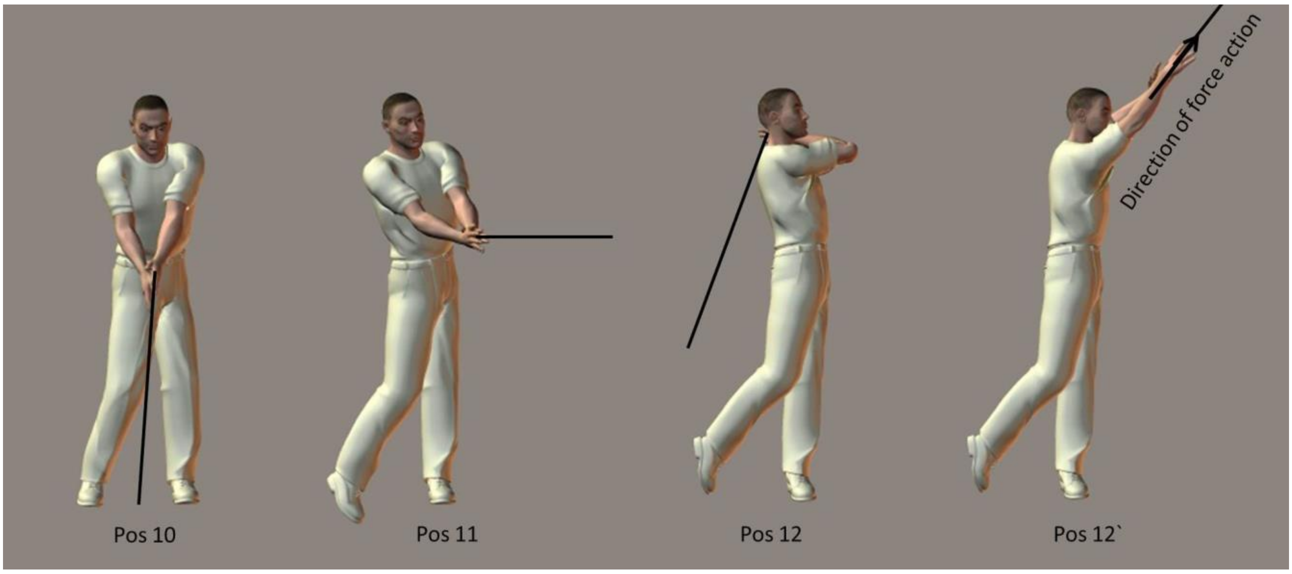


*Figure A6. Frontal view of the finish of the golf swing according to the free-release method (Positions 10-12), and with an alternative final position (Position 12') with a more effective amortisation of the club's kinetic energy in order to reduce flexion strain on the spine*

*Table A4. Functional Assignments for Modalities of the Golf Swing Sub-action "Finish"*

| | Modalities | Functional Assignments |
|---|---|---|
| **a** | **Finishing weight transfer** | |
| a1 | The weight transfer to the left foot is finished after the ball has been hit | In order to avoid blocking the swing, and as a consequence, to be able to realise a joint-careful amortisation of the club's kinetic energy |
| a2 | The axis of body rotation is shifted to the centre of the left foot | In order to avoid blocking the swing, and as a consequence, to be able to realise a joint-careful amortisation of the club's kinetic energy |
| **b** | **Body rotation until finish position** | |
| b1 | The pelvis and the shoulders rotate so that the pelvis and shoulder axes are perpendicular to the target horizon | In order to avoid blocking the swing, and as a consequence, to be able to realise a joint-careful amortisation of the club's kinetic energy |
| b2 | The eyes follow the ball | In order to receive visual feedback of the ball flight in terms of an action effect |
| b3 | The body rotation is accompanied by a head rotation into the same direction | In order to avoid blocking the swing, and as a consequence, to be able to realise a joint-careful amortisation of the club's kinetic energy |
| **c** | **Remaining in finish position** | |
| c1 | The position with the body weight mainly on the left leg is held for approximately 3 s | In order to guarantee high control, and as a consequence, prophylaxis of injury and in order to receive feedback whether the swing has been performed in a balanced fashion, overall |
| c2 | The spine is neither hyper-extended nor flexed sideward by activation of the abdominal muscles | In order to protect the spine against injuries |
| c3 | The shaft of the club is deposited at the neck | In order to achieve an optimal amortisation of the kinetic energy |

Although not explicitly proposed by the free-release method, it should be added that from a functional point of view, an alternative finish position could be substantiated in order to further relieve the spine from strain (see Figure A6, Pos. 12'). This position would not be marked by the commonly used reversed-C posture.

Instead, the arm and the club would be led into a diagonal forward direction over the head. A golf swing ending in such a finish position would pronouncedly reduce flexion strain on the spine because the vector of the resulting force would target into a vertical upward direction. In terms of aesthetics as well as tradition, it could seem rather strange to perform this alternative task solution; however, with respect to preventing injuries, especially in golf as a leisure sports, it seems advisable to consider movement variants that are distinguished by superiority in functional respect.

# References


Ball, K. A., and Best, R. J. (2007). Different centre of pressure patterns within the golf stroke I: Cluster analysis. *J. Sport. Sci.* 25, 757-770.

Batt, M. E. (1992). A survey of golf injuries in amateur golfers. *Brit. J. Sport. Sci.* 26, 63-65.

Beak, S.-H., Choi, A., Choi, S.-W., Oh, S. E., Mun, J. H., Yang, H., and Song, H.-R. (2013). Upper torso and pelvis linear velocity during the downswing of elite golfers. *BioMed. Eng. OnLine* 12, 13.

Brown, S. J., Selbie, W. S., and Wallace, E. S. (2013). The X-factor: An evaluation of common methods used to analyse major inter-segment kinematics during the golf swing. *J. Sport. Sci.* 31, 1156-1163.

Cheetham, P. J., Martin, P. E., Mottram, R. E., and St Laurent, B. F. (2000). The importance of stretching the "X-Factor" in the downswing of golf: The "X-Factor Stretch.". In *Optimising Performance in Golf*, ed. P. R. Thomas (Brisbane/Australia: Australian Academic Press), 192-199.

Chu, Y., Sell, T. C., and Lephart, S. M. (2010). The relationship between biomechanical variables and driving performance during the golf swing. *J. Sport. Sci.* 28*,* 1251-1259.

Cochran, A., and Stobbs, J. (1969). *The Search for the Perfect Swing*. New York: J.P. Lippincott.

Drollinger, F. (2012). *Golf. The New Testament*. Birkenfeld/Germany: Novus Plus.

Fitts, P. M. (1954). The information capacity of the human motor system in controlling the amplitude of movements. *J. Exp. Psychol.* 47, 381-391.

Hetu, F. E., Christie, C. A., and Faigenbaum, A. D. (1998). Effects of conditioning on physical fitness and club head speed in mature golfers. *Percept. Motor Skill.* 86, 811-815.

Hogan, B. (1957). *Five Lessons: The Modern Fundamentals of Golf*. New York: Simon & Schuster.

Hogan, B., and Wind, H. W. (1985). *Five Lessons: The Modern Fundamentals of Golf*. New York: Simon & Schuster.

Hume, P. A., Keogh, J., and Reid, D. (2005). The role of biomechanics in maximising distance and accuracy of golf shots. *Sports Med.* 35*,* 429-449.

Kenny, I., McCloy, A., Wallace, E., and Otto, S. (2008). Segmental sequencing of kinetic energy in a computer-simulated golf swing. *Sports Eng.* 11, 37-45.

Knudson, D. (2007). *Fundamentals in Biomechanics* (2nd ed.). New York: Springer Science.

McHardy, A., Pollard, H., and Luo, K. (2006). Golf injuries: A review of the literature. *Sports Med.* 36, 171-187.

Nesbit, S. M., and McGinnis, R. (2009). Kinematic analyses of the golf swing hub path and its role in golfer/club kinetic transfers. *J. Sport. Sci. Med.* 8, 235-246.

Peper, G., and Tiegreen, M. (2005). *The Secret of Golf: A Century of Groundbreaking, Innovative, and Occasionally Outlandish Ways to Master the World's Most Vexing Game*. New York: Workman.

Plamondon, R., and Alimi, A. M. (1997). Speed/accuracy trade-offs in target-directed movements. *The Behav. Brain Sci.* 20, 279-303.

Sato, K., Kenny, I C., and Dale, B. R. (2013). Current golf performance literature and application to training. *Journal of Trainology* 2, 23-32.

Schneider, K. (1990). *Koordination und Lernen von Bewegungen. Eine experimentelle Bestätigung von Bernsteins Koordinationshypothese* [Coordination and learning of movements. An experimental confirmation of Bernstein's coordination hypothesis]. Frankfurt/Germany: Deutsch.

Sigrist, R., Rauter, G., Riener, R., and Wolf, P. (2013). Augmented visual, auditory, haptic, and multimodal feedback in motor learning: A review. *Psychon. Bul. Rev.* 20, 21-53.

Snead, S., and Stump. A. (1962). *The Education of a Golfer*. New York: Simon and Shuster.

Sugaya, H., Tschiya, A., Moriya, H., Morgan, D. A., and Banks, S. A. (1998). Low-back injury in elite and professional golfers an epidemiologic and radiographic study, in *Science & Golf III. Proceedings of the world scientific congress of golf*, ed. M. R. Farrally and A. J. Cochran (Champaign, IL: Human Kinetics), 83-91.

Torres-Ronda, L., Sanchez-Medina, L., and Gonzalez-Badillo, J. J. (2011). Muscle strength and golf performance: A critical review. *J. Sport. Sci. Med.* 10, 9-18.
